# Midlife work-related stress is associated with late-life cognition

**DOI:** 10.1007/s00415-017-8571-3

**Published:** 2017-08-18

**Authors:** Shireen Sindi, Ingemar Kåreholt, Alina Solomon, Babak Hooshmand, Hilkka Soininen, Miia Kivipelto

**Affiliations:** 1 Aging Research Center, Karolinska Institutet and Stockholm University, Gävlegatan 16, 8th Floor, 113 30 Stockholm, Sweden; 2 Division of Clinical Geriatrics, Center for Alzheimer Research, Karolinska Institutet, Stockholm, Sweden; 3 Neuroepidemiology and Ageing Research Unit, School of Public Health, Imperial College London, London, UK; 4 Institute of Gerontology, School of Health and Welfare, Aging Research Network-Jönköping (ARN-J), Jönköping University, Jönköping, Sweden; 5 Institute of Clinical Medicine, Neurology, University of Eastern Finland, Kuopio, Finland; 6 Department of Geriatrics, Karolinska University Hospital, Stockholm, Sweden; 7 Neurocenter, Neurology, Kuopio University Hospital, Kuopio, Finland; 8 Department of Chronic Disease Prevention, National Institute for Health and Welfare, Helsinki, Finland

**Keywords:** Work-related stress, Stress, Job demands, Job strain, Cognition, Midlife risk factors

## Abstract

To investigate the associations between midlife work-related stress and late-life cognition in individuals without dementia from the general population. The Cardiovascular Risk Factors, Aging and Dementia (CAIDE) study population (*n* = 2000) was randomly selected from independent Finnish population-based surveys (baseline mean age 50 years). Participants underwent two re-examinations in late life (mean age 71 and 78 years, respectively). 1511 subjects participated in at least one re-examination (mean total follow-up 25 years). Work-related stress was measured using two questions on work demands administered in midlife. Multiple cognitive domains were assessed. Analyses were adjusted for several potential confounders. Higher levels of midlife work-related stress were associated with poorer performance on global cognition [*β*-coefficient, −0.02; 95% confidence interval (CI), −0.05 to −0.00], and processing speed [*β* −0.03, CI −0.05 to −0.01]. Results remained significant after adjusting for potential confounders. Work-related stress was not significantly associated with episodic memory, executive functioning, verbal fluency or manual dexterity. This study shows that global cognition and processing speed may be particularly susceptible to the effects of midlife work-related stress.

## Introduction

Job strain is a common and important source of stress; especially considering the large proportion of time individuals spend at work throughout their lifespan. Chronically elevated work-related stress is a well-established risk factor for numerous physical and mental health outcomes, including depression, metabolic syndrome and cardiovascular diseases [5, 20, 26, 28, 33]. More recently, studies have also shown associations between work-related stress and dementia, where high job strain, low levels of job control, low social support at work, and more stress-related physical symptoms have all been associated with higher dementia risk later in life [2, 7, 25, 34]. Consistently, high levels of job control and high challenges were associated with a reduced risk for dementia [24].

Some studies have also investigated the associations between work-related stress and cognition with less than a handful of longitudinal studies simultaneously measuring multiple cognitive domains. Work-related stress in the form of low job control and high job strain was associated with worse cognition and cognitive decline, although the findings have been mixed regarding the compromised cognitive domains. Longitudinal results showed that active jobs (characterized by high levels of both demands and control) were associated with higher performance on a phonemic test of verbal fluency, but not with other cognitive domains after adjustment for employment grade [8]. Similarly, low job control was associated with poor global cognition, whereas active jobs were associated with better global cognition [3, 20]. Low job control and more job strain were also associated with poor episodic memory at retirement, and more rapid episodic memory decline post-retirement [4]. Another study showed that high job strain and low control were associated with decline in verbal learning and memory, but not visual memory, where the tests were administered approximately 15 and 21 years after assessment of job strain [2]. Evidence also showed that job strain and demands impact subjective cognitive complaints and learning outcomes [13, 27, 29]. Taken together, these findings emphasize the importance of measuring multiple cognitive domains using a life-course approach when examining the impact of work-related stress on cognition.

The job demand-control-support model is one of the most common models for conceptualizing work-related stress [15, 30]. According to this model, high job demands, low job control and their combination are associated with various health and cognitive outcomes [19]. It has recently been suggested that more evidence is needed on the effects of self-reported job strain on cognitive outcomes [4]. In this population-based cohort study, we investigated the associations of midlife work-related stress and specifically job demands with late-life cognitive performance in several domains among individuals without dementia (average follow-up time 25 years).

## Materials and methods

### Study population

**Fig. 1:**
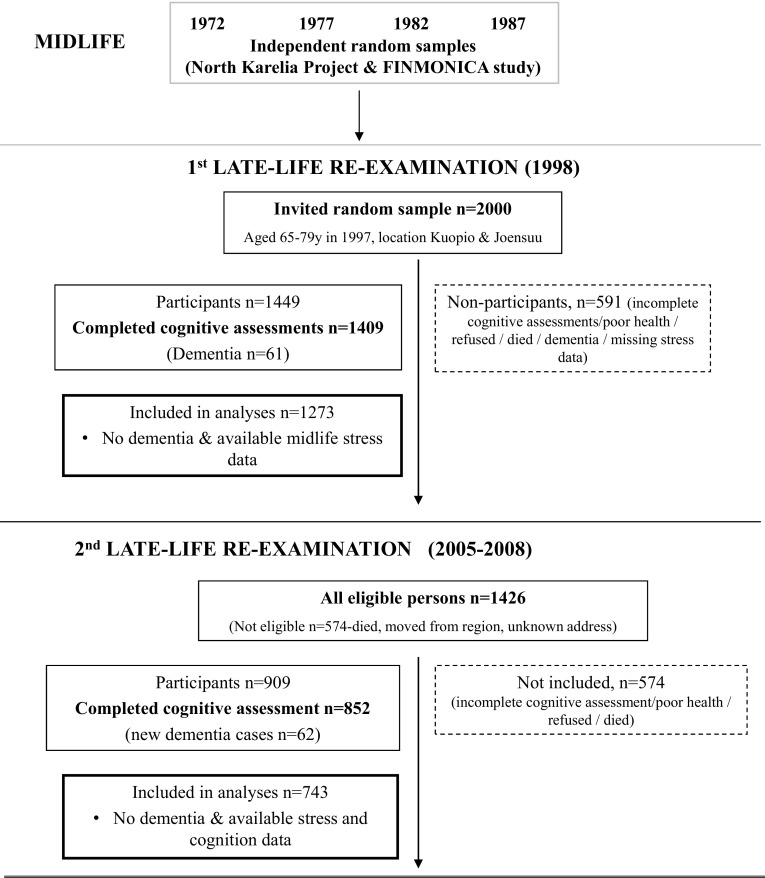
Flowchart representing the study population, examinations and cognitive assessments in the CAIDE study

Participants of the Cardiovascular Risk Factors, Aging and Dementia (CAIDE) study in Finland were first examined at midlife (baseline) in the North Karelia Project and the FINMONICA study, where individuals were assessed in one of the following years for the baseline assessment: 1972, 1977, 1982 or 1987 [22]. Baseline participation rates ranged between 82 and 90%. In 1998, a random sample of 2000 survivors living in the cities of Kuopio and Joensuu, aged 65–79, were invited for a first re-examination (Fig. 1). A total of 1449 (72.5%) individuals participated and 1409 completed the cognitive assessments. The mean follow-up time was 21 years (SD = 4.9). Participants returned for a second re-examination between 2005 and 2008. In 2005, of the 2000 original sample, 1426 were still alive and were still living in the same region. When invited, 909 (63.7%) of them participated and 852 completed the cognitive assessment. A total of 1511 individuals participated in at least one re-examination, and 750 participated in both. Mean ages at each time point were: at baseline, 50 years (SD = 6.0, age range: 39-64); at the first re-examination, 71.3 years (SD = 4.0, age range 65–80); at the second re-examination, 78.6 years (SD = 3.7, age range 72–90). Local ethics committees approved the CAIDE study and participants provided written informed consent. The study complies with the Declaration of Helsinki.

#### Measurement of work-related stress

Perceived work-related stress was measured in midlife using two questions focusing on job demands. These questions were adapted from the questions validated by Karasek et al. [16] and have been used reliably by various research groups [3, 20, 31]. Both questions have the same 5-point likert scale. The questions were, “How often do you struggle to cope with the amount of work?” and “How often are you bothered by constant hurry at work?”. After reverse coding to facilitate the interpretation of the results, the response options were: 1 = never, 2 = rarely, 3 = sometimes, 4 = often, 5 = always. Data on work-related stress were available for 1273 participants. Both questions were highly correlated (Spearmans *ρ* = 0.623, *p* < 0.001) and were summed to produce a composite measure of work-related stress.

#### Cognitive assessments

At both re-examinations, a comprehensive battery of neuropsychological tests was administered to assess multiple cognitive domains. For the current study, we used the following tests that were administered at both re-examinations: (1) global cognition measured by the mini mental state examination (MMSE) [10]; (2) episodic memory assessed by an immediate word recall test (10-word list); (3) executive functioning measured by the Stroop test (time difference between the task of naming the color of the ink used to write the name of another color, and the task of naming colors of dots); (4) verbal fluency tested by category fluency test (number of correct animal names generated in 60 s); (5) psychomotor speed assessed by the letter digit substitution test; (6) manual dexterity measured by the bimanual Purdue Pegboard test. Dementia diagnosis was carried out using a three-step protocol previously described [25].

#### Other assessments

At baseline (midlife), assessments and survey methods were standardized and adhered to international guidelines and the World Health Organization (WHO) (Multinational MONItoring of trends and determinants in CArdiovascular disease) MONICA protocol [21]. Re-examination surveys were similar and comparable to those at baseline. Baseline surveys involved self-administered questionnaires on medical history, sociodemographic factors, health status, health-related behaviors and psychological-related factors. We selected the following covariates previously shown to be associated with worse cognition and/or high levels of stress: age, sex, education, *APOE* ε4, respiratory, cardio/cerebrovascular and musculoskeletal conditions, and type of occupation (white collar vs. blue collar). Occupation type was measured by asking individuals to select their longest-held occupation among the following categories: office/service, farming/forestry, mining/industrial/construction work, housewives, or other. Hopelessness was measured using the following two questions described previously [11]: “I feel that it is impossible to reach the goals I would like to strive for” and “The future seems to me to be hopeless, and I cannot believe that things are changing for the better”. A five-point Likert scale was used, coded as 0 = absolutely agree; 1 = somewhat agree; 2 = cannot say; 3 = somewhat disagree; or 4 = absolutely disagree. A trained nurse verified the answers and addressed participants’ questions. The nurse also measured height, weight and blood pressure. A venous blood sample was obtained, and allowed for measures of biomarkers, including cholesterol and *APOE* genotype from blood leucocytes, for which HHaI digestion and polymerase chain reaction were used [32]. The Hospital Discharge Register was used for information on respiratory and cardio/cerebrovascular conditions (chronic obstructive pulmonary disease, asthma, coronary artery disease, stroke, myocardial infarction, atrial fibrillation, cardiovascular surgery, heart failure or diabetes). These conditions were combined into a dichotomous variable (yes/no) reflecting the presence of any midlife respiratory or cardio/cerebrovascular conditions. All covariates were measured at baseline.

### Statistical analyses

We conducted analyses using Stata 13.0 (Stata Corp, College Station, TX, USA). We analyzed participant baseline characteristics using Chi-square (*χ* ^2^) tests for categorical variables (data reported as percentages), and Student *t* tests for continuous variables (data reported as means (standard deviations [SD])). The significance level for all analyses was set at *p* < 0.05 (Table 1). Zero-skewness log-transformations were applied to cognitive test scores (Stata command lnskew0). Results were standardized to have SD = 1.

**Table 1:** Sociodemographic and clinical characteristics of participants included in the analyses at the first and second re-examinations

Characteristics	First re-examination (1998)		Second re-examination (2005–2008)	
	*n*	Mean (SD) or *n* (%)	*n*	Mean (SD) or *n* (%)
Baseline age	1273	49.9 (5.9)	743	49.0 (5.7)
Age at follow-up	1273	71.0 (4.0)	743	78.3 (3.6)
Follow-up time	1273	21.1 (4.8)	743	29.3 (4.9)
Sex				
Women	1273	791 (62.1)	743	482 (64.9)
Education (years)	1255	8.8 (3.4)	733	9.2 (3.4)
*APOE*ε4 allele				
Carrier	1247	437 (35.0)	659	203 (30.8)
Work-related stress (range 0–8)	1273	3.4 (1.9)	743	3.4 (1.9)
Type of occupation				
White collar	1231	603 (49.0)	719	381 (53.0)
Other	1231	628 (51.0)	719	338 (47.0)
Midlife cardio/cerebrovascular/respiratory conditions				
Yes	1235	38 (3.0%)		15 (2.0%)

Column wise values are numbers (%), and *χ* ^2^ test was used. Values are means (SD)

Participants with dementia at the first re-examination were excluded from analyses at the first re-examination. Participants with dementia at the second re-examination were excluded from analyses at the second re-examination. To maximize sample size, all subjects with cognitive assessments in at least one re-examination (*n* = 1332) were considered in analyses. This means that the analyses are based on both the first and second re-examination combined. For subjects with cognitive assessments in both re-examinations (*n* = 685), two observations were included (i.e., one for test results at the first re-examination, and one for test results at the second re-examination). Data were organized in what is often referred to as long format.

To investigate the associations between midlife work-related stress and cognition, we performed linear regression analyses for each of the cognitive domains. We reported results as *β*-coefficient and 95% confidence intervals (CI). All analyses were adjusted for a basic set of confounders: age, sex, years of education and follow-up time (Model 1). Model 2 additionally adjusted for the type of occupation. Model 3 additionally for *APOE* ε4 genotype, hopelessness and midlife respiratory, cardio/cerebrovascular conditions.

## Results

### Population characteristics

Table 1 shows sociodemographic and clinical characteristics of the participants included in the analyses. Table 2 shows the raw scores for all the cognitive tests administered at the first and second re-examinations. In this sample, 76 individuals were diagnosed with mild cognitive impairment at the first re-examination. Of them, 27 were alive at the second re-examination, and 6 of them converted to dementia (for details regarding the mild cognitive impairment diagnoses, see [25]). A total of 156 had mild cognitive impairment at the second re-examination. The dementia cases were excluded from analyses.

**Table 2:** Descriptive statistics of cognitive test scores at the first and second re-examinations

Characteristics	First re-examination (1998)		Second re-examination (2005–2008)	
	*n*	Median (range)	*n*	Median (range)
Global cognition (Mini Mental State Exam)	1273	26 (20–30)	743	27 (20–30)
Episodic memory (word list recall)	1271	5 (0–10)	742	5 (1–10)
Executive functioning (Stroop)	1212	36 (1–257)	726	40 (7–250)
Verbal fluency	1268	20 (9–55)	742	20 (8–40)
Letter digit substitution	1228	19 (4–50)	655	20 (9–47)
Purdue Peg Board	1209	10 (4–18)	727	8 (1–13)

### Associations between work-related stress and cognition

Associations between midlife work-related stress and cognition are shown in Table 3.

**Table 3:** The associations between midlife work-related and late-life cognition

	Model 1	Model 2	Model 3
Cognitive domain (test)	*β*-coefficient (95% CI)	*β*-coefficient (95% CI)	*β*-coefficient (95% CI)
Global cognition (Mini Mental State Exam)	−0.02 (−0.04 to 0.00)	−0.02 (−0.04 to −0.00)	−0.02 (−0.04 to −0.00)
Episodic memory (word list recall)	−0.01 (−0.03 to 0.01)	−0.01 (−0.03 to 0.01)	−0.00 (−0.02 to 0.02)
Executive functioning (Stroop)	−0.00 (−0.03 to 0.02)	−0.00 (−0.02 to 0.02)	0.00 (−0.02 to 0.03)
Verbal fluency (animal naming)	−0.02 (−0.04 to 0.00)	−0.02 (−0.04 to 0.00)	−0.01 (−0.03 to 0.01)
Processing speed (letter digit substitution)	−0.02 (−0.04 to −0.00)	−0.03 (−0.05 to −0.01)	−0.03 (−0.05 to −0.01)
Manual dexterity (Purdue Peg Board)	−0.01 (−0.03 to 0.02)	−0.01 (−0.03 to 0.02)	−0.01 (−0.03 to 0.01)

Model 1: age, sex, follow-up time, education

Model 2: Model 1 + occupation type

Model 3: Model 2 + *APOE*4, midlife hopelessness and midlife cardio/cerebrovascular/respiratory conditions

Based on data in long format, individuals with observations at both re-examinations each contribute to two observations

Higher levels of work-related stress were significantly associated with worse global cognition measured by the MMSE after the adjustment for occupation type (Model 2: *β* −0.02, 95% CI −0.04 to −0.00) , APOEε4, hopelessness and midlife cardio/cerebrovascular/respiratory conditions (Model 3: *β* −0.02, 95% CI −0.05 to −0.00).

Higher levels of work-related stress were also associated with poorer performance on processing speed measured by the letter digit substitution test in all three models (Model 1: *β* −0.02, 95% CI −0.04 to −0.00; Model 2: *β* −0.03, 95% CI −0.05 to −0.01; Model 3: *β* −0.03, 95% CI −0.05 to −0.01). Work-related stress was not significantly associated with episodic memory, verbal fluency, executive functioning or manual dexterity (all *p* > 0.05).

## Discussion

This study shows that midlife work-related stress characterized by high job demands and constant hurry at work is associated with poorer performance on global cognition, and lower performance on processing speed, in a large representative population without dementia. These associations remained significant after adjusting for several confounding factors. In contrast, work-related stress was not significantly associated with episodic memory, verbal fluency, executive functioning or manual dexterity.

The current study supports previous findings showing that job strain and low job control are associated with worse MMSE performance [3, 20]. Our results are inconsistent with results from the Framingham study, where job strain and low job control were associated with verbal learning and memory [2], and results from the Health and Retirement Study showing that job strain expressed as low job control was associated with poor episodic memory performance at retirement, and further decline after retirement [4]. These discrepancies may be due to different measures of work-related stress across the studies, the different follow-up durations, the different cognitive tests (immediate vs. delayed recall) and geographical differences between work-related stress levels between the USA and Nordic or other European countries.

In the current study, the associations between work-related stress and cognition were specific to a few cognitive domains, and did not extend to all measured domains. In the Framingham study, findings were also limited to verbal learning and memory, and not visual memory or abstract reasoning [2]. Similar to the interpretation of Eloviainio et al., the long duration between work-related stress and the assessment of cognitive function (assessed after retirement), may have led to a “dilution of the effects” [8]. Our study adds to the few longitudinal studies with a long follow-up duration, as it is the first to show significant associations with processing speed, in addition to the previously observed association with global cognition assessed by the MMSE.

Several mechanisms may underlie the observed associations. First the stress hormone cortisol has its receptors in brain regions involved in learning, memory and executive functioning [12]. Previous evidence has shown that higher cortisol levels are associated with worse MMSE and processing speed performance [6, 17]. Although our findings are inconsistent with studies on cortisol levels and worse episodic memory performance, these differences may be due to the different time durations between stress exposure and cognitive assessment, or the sensitivity of cognitive tasks used [17, 18]. We analyzed immediate recall. If we instead had analyzed delayed recall, we may have found an association. Another mechanism is through elevations in allostatic load, a multi-system measure of cumulative stress that has also been previously associated with poor cognition [14].

Work-related stress may be reduced through various interventions, some of which may target individuals, by providing social support, recognition, increasing the sense of control over work-related tasks, offering constructive feedback as well as professional development opportunities [9, 23]. Indeed, higher social support at work is associated with fewer cognitive complaints and reduces the risk for dementia [27]. The work environment can also be ameliorated by improving the work climate, the physical work environment and increasing cooperation/teamwork [9, 23]. As previously suggested by Andel et al., interventions may also target retired older adults who had jobs characterized by low control, and we add to that suggestion ‘job strain’, to prevent cognitive decline [4].

Our study has several strengths including the long follow-up duration from midlife to later life, the large population, measurement of cognitive performance using several validated tests for different cognitive domains, and adjusting for potential confounders. The study also has some limitations. First, stress in later life and non-work-related stress were not measured, so it is unclear whether the role of midlife work-related stress is independent of these other sources of stress. Second, although job control is an important dimension of work-related stress, it was not measured in the current study [24, 34].

In conclusion, our study shows that midlife work-related stress is associated with worse global cognition and processing speed performance. These findings suggest that the effects of work-related stress are long lasting, with some cognitive domains more sensitive than others.
